# Fish scales and SNP chips: SNP genotyping and allele frequency estimation in individual and pooled DNA from historical samples of Atlantic salmon (*Salmo salar*)

**DOI:** 10.1186/1471-2164-14-439

**Published:** 2013-07-03

**Authors:** Susan E Johnston, Meri Lindqvist, Eero Niemelä, Panu Orell, Jaakko Erkinaro, Matthew P Kent, Sigbjørn Lien, Juha-Pekka Vähä, Anti Vasemägi, Craig R Primmer

**Affiliations:** 1 Department of Biology, University of Turku, Turku FIN-20014, Finland; 2 Finnish Game and Fisheries Research Institute, Utsjoki FIN-99980, Finland; 3 Centre for Integrative Genetics (CIGENE), Department of Animal and Aquacultural Sciences, Norwegian University of Life Sciences, Aas N - 1432, Norway; 4 Department of Aquaculture, Estonian University of Life Sciences, Kreutzwaldi 48, 51014, Tartu, Estonia; 5 Institute of Evolutionary Biology, University of Edinburgh, Edinburgh EH9 3JT, United Kingdom

**Keywords:** Atlantic salmon, SNP genotyping, Illumina® iSelect SNP-array, Degraded DNA, Archived samples, Fish scales, DNA pooling, Allelotyping, Allele frequency, Fragment size

## Abstract

**Background:**

DNA extracted from historical samples is an important resource for understanding genetic consequences of anthropogenic influences and long-term environmental change. However, such samples generally yield DNA of a lower amount and quality, and the extent to which DNA degradation affects SNP genotyping success and allele frequency estimation is not well understood. We conducted high density SNP genotyping and allele frequency estimation in both individual DNA samples and pooled DNA samples extracted from dried Atlantic salmon (*Salmo salar*) scales stored at room temperature for up to 35 years, and assessed genotyping success, repeatability and accuracy of allele frequency estimation using a high density SNP genotyping array.

**Results:**

In individual DNA samples, genotyping success and repeatability was very high (> 0.973 and > 0.998, respectively) in samples stored for up to 35 years; both increased with the proportion of DNA of fragment size > 1000 bp. In pooled DNA samples, allele frequency estimation was highly repeatable (Repeatability = 0.986) and highly correlated with empirical allele frequency measures (Mean Adjusted R^2^ = 0.991); allele frequency could be accurately estimated in > 95% of pooled DNA samples with a reference group of at least 30 individuals. SNPs located in polyploid regions of the genome were more sensitive to DNA degradation: older samples had lower genotyping success at these loci, and a larger reference panel of individuals was required to accurately estimate allele frequencies.

**Conclusions:**

SNP genotyping was highly successful in degraded DNA samples, paving the way for the use of degraded samples in SNP genotyping projects. DNA pooling provides the potential for large scale population genetic studies with fewer assays, provided enough reference individuals are also genotyped and DNA quality is properly assessed beforehand. We provide recommendations for future studies intending to conduct high-throughput SNP genotyping and allele frequency estimation in historical samples.

## Background

Historical archived samples are an important resource for population genetic monitoring, as they allow us to understand the impact of anthropogenic influences and environmental change in the wild [1]. Viable DNA obtained from historical material such as museum specimens, scales, feathers, hair and/or bones [2] has been used to examine past population structure [3,4] and its persistence over time [5,6], population collapses [7] and bottlenecks [8], founder events [9] and the consequences of stocking of populations with non-native individuals [10]. However, a crucial limitation of historical samples is that they generally yield DNA of a lower amount/quality than is recommended for molecular genetic studies. This is due to a number of factors, including: DNA degradation over long-term storage in sub-optimal conditions; sample age; the sample quality at initial sampling; the relative DNA concentration within the sample; and the reduced efficiency of DNA extraction protocols on the sample type. All of these factors can lead to a risk of PCR failure, genotyping errors and allelic drop-out [11,12], as well as a failure to fulfil recommendations regarding the amount and quality of DNA used for genotyping.

With the advent of next generation sequencing and cost-efficient genotyping technology, single-nucleotide polymorphisms (SNPs) are an increasingly popular molecular marker in genetic and evolutionary research. They occur at higher frequencies throughout the genomes of a wide range of species [13,14] and have the potential to identify functionally important polymorphisms [15,16]. Compared to using more traditional markers, such as microsatellites, SNP genotyping is faster, more cost-efficient and less error-prone when considering the assessment of thousands, rather than tens of loci [17,18]. This is because it can be carried out using low density arrays [11,19] and/or high throughput chips [20]. Furthermore, it is possible to estimate population-wide allele frequencies in pooled DNA samples within a single SNP array, meaning that population genetic studies and outlier analyses could be conducted using a considerably reduced number of assays [21-24]. Consequently, SNP markers have an enormous potential to address a number of outstanding questions in evolutionary ecology, conservation genetics and wildlife management [25-27] and indeed, encouraging examples of studies utilising SNPs in historical samples are emerging in a number of species [4,28-30].

At present, there are two principle technologies available for genotyping thousands of SNP loci simultaneously in custom arrays (also known as ‘SNP chips’); Illumina Infinium (San Diego, California, USA) [31] and Affymetrix Axiom (Santa Clara, California, USA) [32]. The instrumentation and array construction of the two technologies are very different, but the basic principles of the assay chemistry are similar, and both systems cluster genotypes based on the intensity of the signal and the contrast between the signals from the two alleles of the SNP. Both platforms offer some advantages for genotyping historical samples; in particular, the length of the DNA fragment size required for SNP genotyping on both platforms is small (e.g. 25-100 bp) compared to the length of most microsatellite markers (> 100 bp); indeed SNP genotyping can be more successful than microsatellite genotyping in historical samples [33]. However, there is also potential for some bias as a result of DNA degradation: protocols for both arrays require whole-genome amplification of DNA samples prior to genotyping [31,34], a process which is sensitive to DNA degradation [35]. In addition, studies assessing the usefulness of pooled DNA samples for allele frequency estimation on either platform have only used high quality DNA [21-24]. Therefore, it remains imperative that detailed characterisation of the extent of DNA degradation and stringent pilot testing of SNP typing on either platform in historical samples is carried out before subsequent scientific conclusions and management decisions are made [11].

In this study, we tested the efficacy of SNP genotyping and allele frequency estimation in historical samples from Atlantic salmon (*Salmo salar*). The economic importance of both farmed and wild Atlantic salmon has led to the development of a high throughput custom Illumina® iSelect SNP-array, which consists of 5568 SNPs throughout its genome [36]. In addition, there has been wide-spread sampling of Atlantic salmon scales and/or otholiths for age determination and population monitoring purposes [12]. Both resources provide an excellent foundation for temporal genetic studies examining the evolutionary impact of anthropogenic effects on wild stocks, but require validation that DNA extracted from archived material before it can be used reliably with the developed array. We tested the reliability of SNP genotyping in historical samples using DNA extracted from scale samples that had been collected from wild adult Atlantic salmon over a thirty year period. We address two important questions in the use of SNP genotyping in historical samples: first, how call rate and repeatability of SNP genotyping varies with sample age and DNA quality (using Dataset 1, see below); and second, what is the accuracy of allele frequency estimation in DNA pools created using historical samples (using Dataset 2). We then discuss the application of our findings and provide recommendations for future SNP genotyping studies in historical samples.

## Results

Scale samples were genotyped at 5568 SNP loci using a modified version of the custom-designed Illumina® iSelect SNP-array described previously [36,37] and individual genotypes were scored using the clustering algorithm implemented in the Illumina® GenomeStudio Genotyping Analysis Module v2011.1. As a result of historical genome duplication in salmonids [38], some SNP markers show polyploidy and have been classified as multi-site variants [37]. Therefore, we retained 5317 loci falling within three categories: ‘SNP’ (segregates as a normal diploid SNP, N = 3928), ‘MSV-3′ (where a SNP exists on a single paralogue; N = 873) and ‘Mono’ (N = 516, where loci in Lien et al. 2011 were monomorphic).

### Dataset 1: Temporal variation of DNA quality and genotyping success in archived scale samples

DNA was extracted from archived scales selected from fish captured in the same river tributary in the years 1976, 1987, 1996 and 2006 (4 fish per year, with two independent extractions per fish and two independent genotyping runs per extracted sample) and normalised to 50 ng/μl. The relative concentrations of DNA of different fragment size ranges were then determined for each extraction.

**Figure 1:**
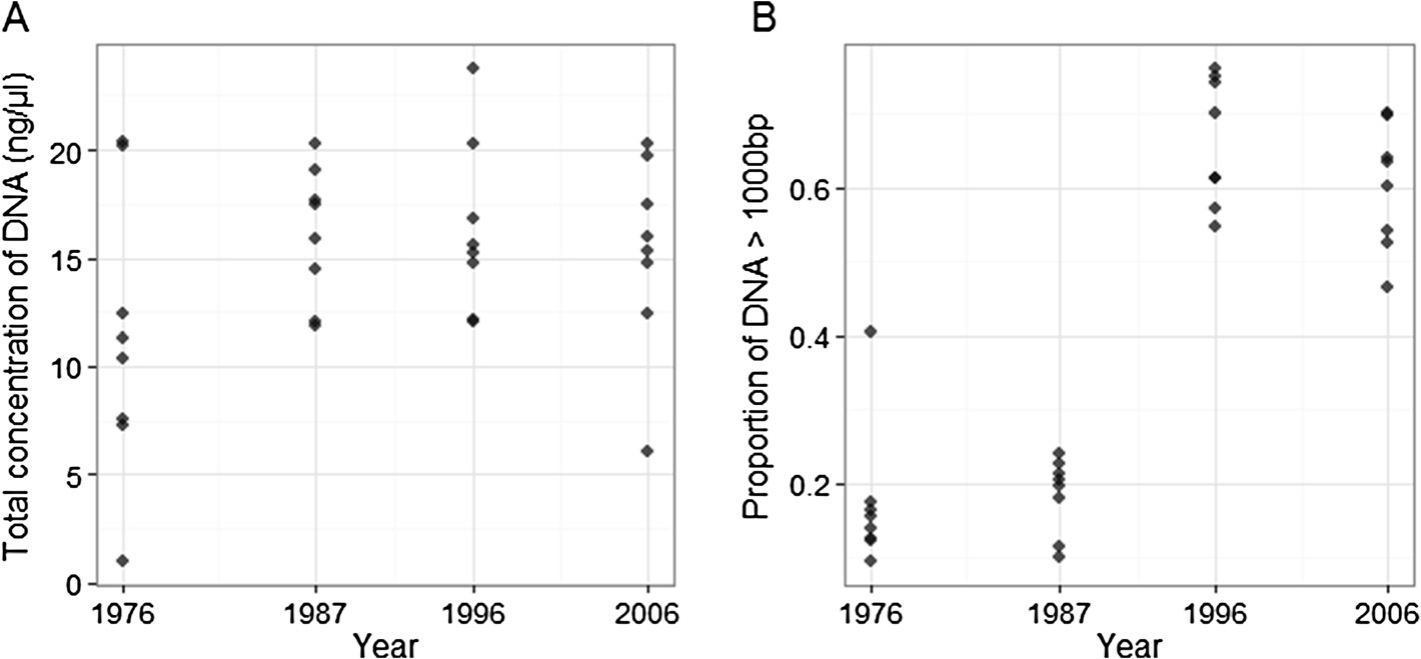
**Temporal variation of DNA quality in archived scale samples.** We show relationships between **A**. Total DNA concentration and Year, and **B**. Proportion of high molecular weight DNA (> 1000 bp) and year. Each point indicates an individual DNA extraction. Samples had been normalised to 50 ng/μl based on NanoDrop Spectrophotometer before total DNA concentration and fragment sizes were measured using an Agilent 2100 Bioanalyzer.

**Figure 2:**
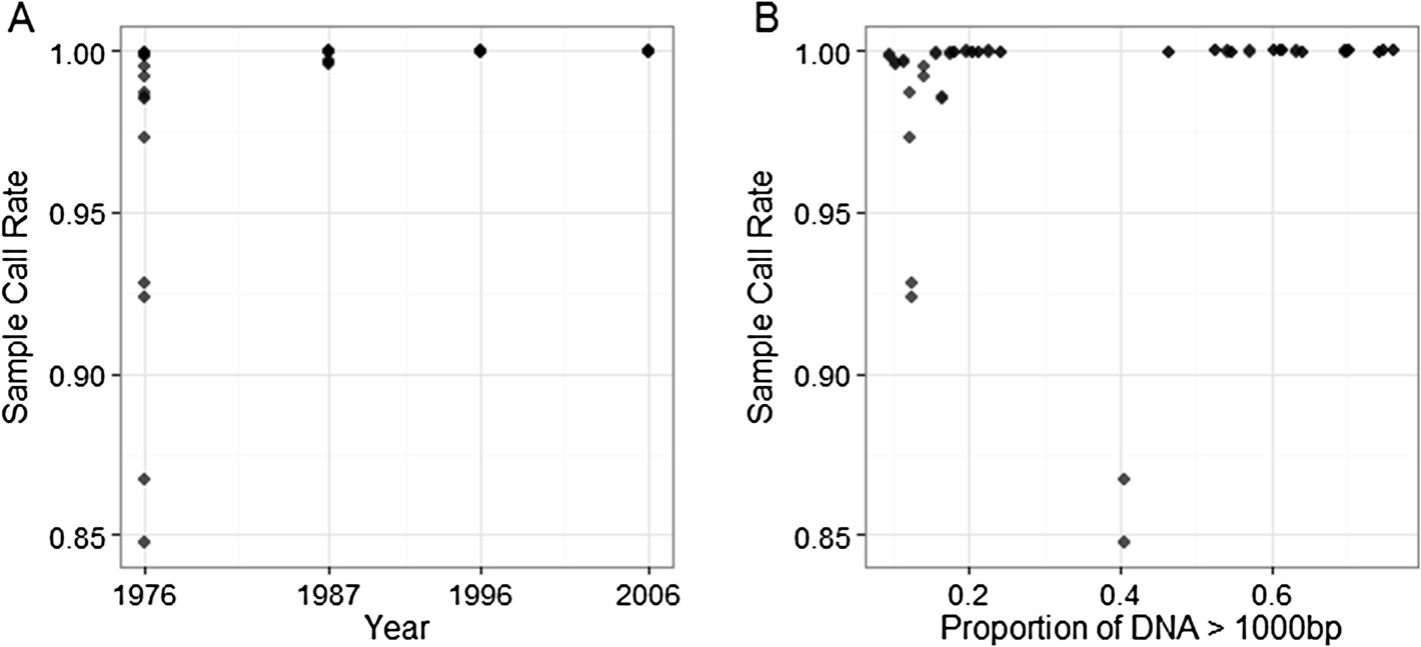
**Temporal variation of genotyping success in archived scale samples.** We show relationships between **A**. Sample Call Rates and Year, and **B**. Sample call rate and proportion of DNA > 1000 bp. Each point indicates an individual genotyping run.

There was no relationship between year and total DNA concentration after normalisation (ρ = 0.242, P = 0.182, Figure 1A), but the proportion of DNA consisting of larger fragment sizes (> 1000 bp) increased with year (ρ = 0.772, P < 0.001, Figure 1B). A total of 4102 loci (including 341 MSV-3 and 377 Mono loci) passed visual inspection and quality control, accounting for 86.2%, 39.1% and 73.1% of SNP, MSV-3 and Mono loci, respectively; 3238 of these loci were polymorphic, with a minor allele frequency (MAF) of > 0.05. Across all years, genotyping rate and the proportion of repeatable genotypes per individual were very high (> 0.973 and > 0.998 across all samples, respectively), with a mean GenCall score (a measure of the reliability the genotype call based on its position relative to the centre of the genotype cluster) of > 0.666. The exception came from samples of one fish sampled in 1976 (ID Ss_1976_011), with values of > 0.848, 0.989 and > 0.530, respectively. Sample call rate also increased with year (ρ = 0.739, P < 0.001, Figure 2A) and with the proportion of DNA with a fragment size of > 1000 bp (ρ = 0.739, P < 0.001, Figure 2B). The full results for all statistical comparisons are given in Additional file 1 and the raw data used to conduct the analysis is provided in Additional file 2.

### Dataset 2: Accuracy of allele frequency estimation (allelotyping) in pooled DNA samples

DNA was extracted from archived scales from 530 fish captured in a large river system between 2001 and 2003 and normalised to 100 ng/μl. Individuals were assigned to one of four DNA pools (N = 87–161 per pool), and genotyping was then carried out on all individual and pooled DNA samples. The ‘empirical’ allele frequency of each locus with each pool was estimated from the individual genotypes of its constituent individuals, and the ‘estimated’ allele frequency for each locus in each pooled DNA sample was determined by its allelic intensity ratio (Theta) relative to the mean theta of each genotype (determined from all individually typed samples; (Additional file 1: Figure S1).

#### Comparison of empirical and estimated allele frequencies

**Figure 3:**
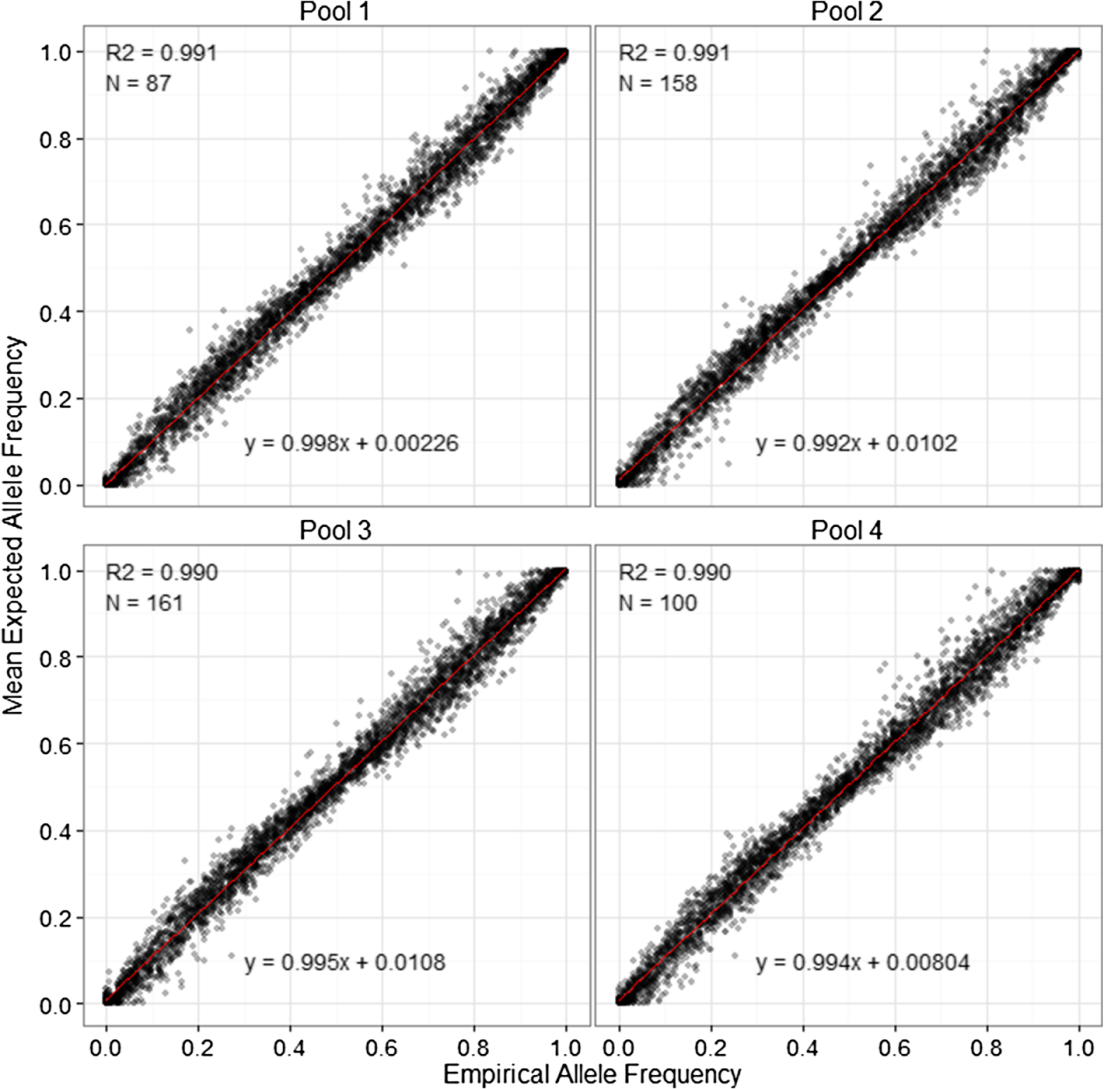
**Correlation between mean estimated allele frequencies and empirical allele frequencies within each pool.** Means were calculated from nine replicates within each pool. R2 is the adjusted R^2^ values from a linear regression. N is the number of individuals included in each pool.

**Figure 4:**
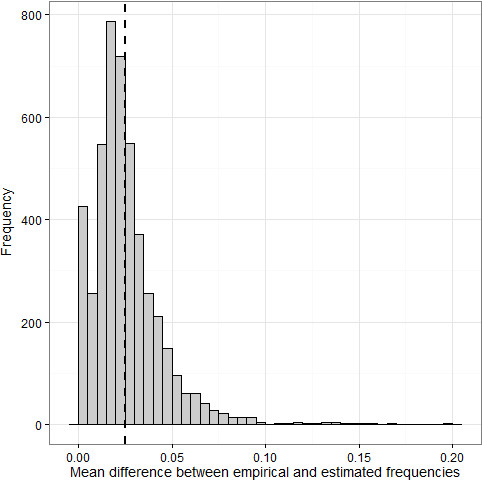
**Histogram of the mean difference between the empirical and estimated allele frequencies for each locus.** The vertical dotted line indicates the mean of the distribution (0.0253).

A total of 4642 loci (including 663 MSV-3 and 460 Mono loci) in 514 individuals passed visual inspection and quality control, accounting for 89.6%, 75.9% and 89.1% of loci categorised as SNP, MSV-3 and Mono, respectively; 3732 of these loci were polymorphic (MAF > 0.05). The correlation between the empirical and estimated allele frequencies for all valid loci was very high across all pools (N = 36; mean adjusted R^2^ = 0.991, SE = 2.121 × 10^-4^; Figure 3) and the mean difference between the empirical and estimated allele frequencies averaged for each locus was 0.0253 (SE = 2.665 × 10^-4^; Figure 4). Higher MAF and lower GenTrain scores (i.e. a measure of clustering efficiency calculated in GenomeStudio) were associated with larger differences between the empirical and estimated values, respectively (General linear model, P < 0.001, Table 1) and loci classified as MSV-3 and Mono had significantly smaller differences between empirical and estimated frequencies compared to SNP loci (P < 0.001). Estimated allele frequencies were highly repeatable across all the replicated measures (Analysis-wide repeatability = 0.986, 95% credible interval = 0.986 - 0.987).

**Table 1:** Results of a general linear model testing factors affecting of allele frequency estimation

**Pooled individual number**	**Model terms**	**Parameter estimate**	**S.E.**	**t-value**	**P-value**
**514 Individuals**	Minor Allele Frequency	0.0211	0.00180	11.75	< 0.001
(**N**_**LOCI**_ = **4642**)	GenTrain Score	−0.0226	0.00196	−11.49	< 0.001
	**Locus Classification**:				
	Mono	−0.0149	0.00094	−15.74	< 0.001
	MSV-3	−0.0045	0.00102	−4.401	< 0.001
**20 individuals**	Minor Allele Frequency	0.0199	0.00352	5.65	< 0.001
(**N**_**LOCI**_ = **4571**)	GenTrain Score	−0.0227	0.00409	−5.55	< 0.001
	**Locus Classification**:				
	Mono	−0.0150	0.00185	−8.14	< 0.001
	MSV-3	0.0063	0.00186	3.37	< 0.001
**50 individuals**	Minor Allele Frequency	0.0093	0.00346	2.68	0.00735
(**N**_**LOCI**_ = **4681**)	GenTrain Score	−0.0302	0.00407	−7.43	< 0.001
	**Locus Classification**:				
	Mono	−0.0145	0.00182	−7.96	< 0.001
	MSV-3	−0.0035	0.00196	−1.79	0.0730

The effects of minor allele frequency, GenTrain score and locus classification on the accuracy of allele frequency estimates were tested based on the mean difference between empirical and estimated allele frequencies per locus for all individuals. Estimated allele frequencies were estimated from clusters of all individuals (N = 514) and random subsets of 20 and 50 individuals. The mean difference in allele frequency was used as a dependent variable with a Gaussian error structure. S.E. is the standard error, t-value is the t statistic value and P-value is the corresponding significance of the model term.

#### Estimation of allele frequencies from sampled subsets of individuals for reference genotypes

**Figure 5:**
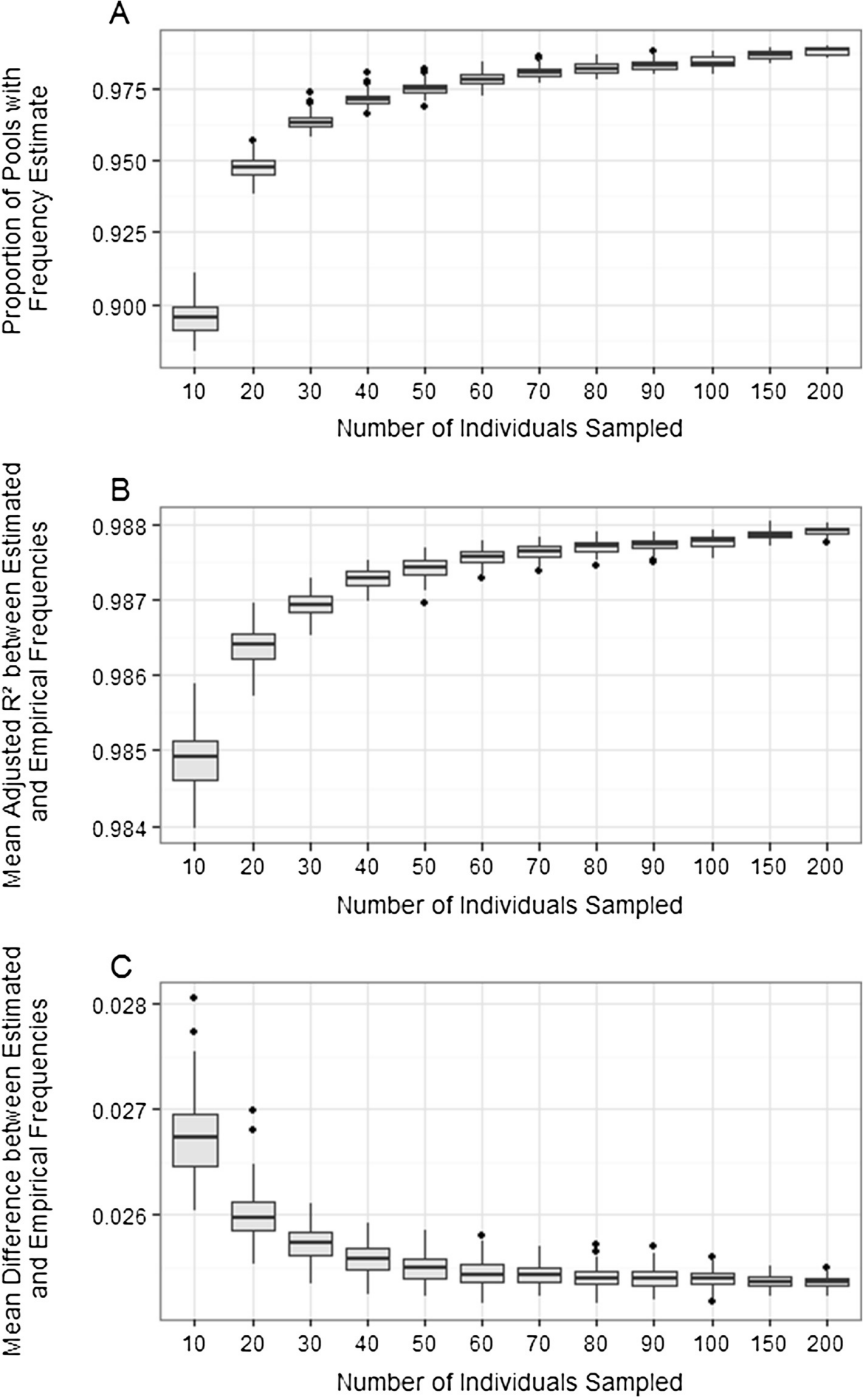
**Boxplot demonstrating the accuracy of allele frequency estimation using subsets of reference individuals.** Parameter estimation was carried out 100 times for each subset. **A**. The proportion of pooled samples for which frequency estimates could be calculated. **B**. The mean adjusted R^2^ over all pools and all loci for each simulation. **C**. The mean difference between empirical and estimated allele frequencies over all pools and loci for each simulation.

Allele frequencies in DNA pools were re-estimated using mean genotype cluster positions calculated from smaller subsets of constituent individuals, ranging from 10 to 200 individuals sampled from the full dataset. The mean proportion of pool allele frequencies that could be estimated increased from 0.895 when estimated from N = 10 individuals, to 0.988 when estimated from N = 200 individuals; a proportion of > 0.95 was observed when sampling 30 individuals or more (Figure 5A). The mean adjusted R^2^ between the empirical and estimated frequencies was high for all subsets (> 0.985) and increased with the number of individuals sampled (Figure 5B). The mean difference between the estimated and empirical allele frequencies decreased as the number of sampled individuals increased (Figure 5C). For all three estimates carried out, the mean values from each subset size were significantly higher from the previous category as the sample size increased (two sample t-test P < 0.001; Figure 5). The mean results obtained for each estimate are given in Additional file 1: Table S4.

#### Re-clustering, genotyping and allele frequency estimation using subsets of representative individuals

**Figure 6:**
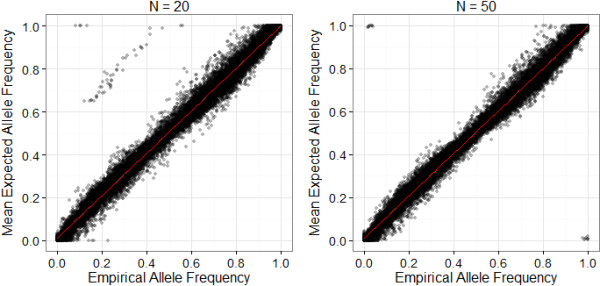
**Correlation between empirical and estimated allele frequencies calculated from 20 (left) and 50 (right) individuals.** Each point represents the mean allele frequency calculated from nine replicates within each of the four pools (= four points per locus). Empirical allele frequencies were determined from the full dataset (N = 514). R2 is the adjusted R^2^ value from a linear regression (red line). NB. Note that some points are far removed from the regression line; these are cases where clusters in duplicated regions of the genome have been placed incorrectly due to the small number of reference individuals.

The entire analysis of pooled samples in Dataset 2 was repeated (from GenomeStudio clustering, genotype determination and visual examination, and allele frequency estimation relative to genotype clusters) using two single subsets of 20 and 50 individuals as reference individuals. Findings were compared to the empirical values determined from the full Dataset 2 (N = 514). In the N = 20 dataset, 4863 loci passed visual inspection and quality control, accounting for 94.0%, 83.8% and 85.3% of loci categorised as SNP, MSV-3 and Mono, respectively; 3738 loci were polymorphic (MAF > 0.05). A total of 111 loci had at least one genotype mismatch when compared to the full dataset (N = 514). Interestingly, 67 of these loci mismatched at > 95% of genotypes, 64 of which had been classified as MSV-3 and Mono loci, indicating that incorrect cluster positioning may be more acute in duplicated regions when using a smaller number of reference individuals. There was a high correlation between the empirical and mean estimated allele frequencies per pool (N_LOCI_ = 4571, mean adjusted R^2^ = 0.978 and SE = 1.806 × 10^-4^; Figure 6A), and the mean difference between the empirical and estimated allele frequencies averaged for each locus was 0.0287 (SE = 4.856 × 10^-4^).

In the N = 50 dataset, 4897 loci passed visual inspection and quality control, accounting for 95.9%, 77.0% and 89.0% of loci categorised as SNP, MSV-3 and Mono, respectively; 3882 loci were polymorphic (MAF > 0.05). Of these, 59 loci had at least one genotype mismatch with the full dataset (N = 514), with 35 loci mismatching at >95% of genotypes (33 of these classified as ‘MSV3′ or ‘Mono’). The increase in sample size resulted in a higher correlation between the empirical and mean estimated allele frequencies per pool (N_LOCI_ = 4681; mean adjusted R^2^ = 0.980, SE = 2.198 × 10^-4^; Figure 6B) and lower mean difference between the empirical and estimated allele frequencies averaged for each locus was 0.0267 (SE = 4.785 × 10^-4^). In both the N = 20 and N = 50 datasets, higher minor allele frequencies and lower GenTrain scores were associated with larger differences between the empirical and estimated values, respectively (General linear model, P < 0.001, Table 1); however, in the N = 20 dataset, MSV-3 loci had a significantly larger differences in between the empirical and estimated allele frequencies (P = 7.51 × 10^-4^).

## Discussion

### DNA fragment profiling in archived samples (Dataset 1)

After normalisation, the total concentration of DNA extracted from air-dried scale samples did not vary over time, but the Bioanalyzer DNA concentrations were consistently much lower than the concentration estimated by the NanoDrop Spectrophotometer (a mean of 14.76 μl compared to the expected 50 ng/μl), indicating the NanoDrop method may over-estimate the DNA concentration in degraded DNA samples. There were marked differences in the proportion of DNA comprised of smaller fragment sizes between the two earlier sampling periods (1976 and 1987) compared to the later sampling periods (1996 onwards; Figure 1).

### Genotyping in archived scale samples (Dataset 1)

The genotyping call rate and error rates in the 1987 samples were comparable to the 1996 and 2006 samples, yet the fragment sizes in the 1987 samples were more similar to the 1976 samples. The sample call rate and error rate were more variable in the 1976 samples, with one sample showing a particularly low call rate and higher genotype mismatch rate. In a larger scale study, samples such as this one could be discarded during quality control, but it is important to note that even in this sample, 3432 of the 4102 loci assessed gave scoreable and repeatable genotypes. Furthermore, the remaining samples from 1976 (call rates > 97.3% and mismatch rates of less than 0.2%) show that reliable genotyping is possible in samples up to 35 years old in this case. There was a significant trend for call-rate and genotype mismatch to be correlated (Additional file 1: Figure S6), indicating that lower call rates are a useful guide for excluding potentially unreliable samples. However, in our dataset, this trend is strongly influenced by the single, poorly performing sample, thus it would be recommendable for future research to focus on a more detailed investigation of the robustness of this trend in a larger number of older samples. It is not clear why there are differences in genotyping efficiency between 1976 and 1987 samples when their fragment size proportions were highly similar (Additional file 1: Figure S2). It may be that additional chemical, physical or biological factors other than fragment size affect genotyping success, possibly through inhibition of PCR or direct effects on the structure of the DNA.

### Genotyping success in polyploid loci (Datasets 1 and 2)

The success of genotyping diploid SNP loci was high, with more than 86% of loci providing scoreable genotypes and allele frequency estimates in both datasets. However, in Dataset 1, less than 40% of duplicated MSV-3 loci passed visual inspection, where individual clusters for each genotype could not be determined. As these loci have a smaller range of theta (~0.5, compared to 1 in ’SNP’ loci), poorly defined clusters are more likely to overlap, leading to a larger number undefined genotypes for individual samples. This problem is likely to have been more acute in Dataset 1 for several reasons. First, although 64 samples were genotyped in Dataset 1, these only comprised of 16 individuals. This is likely to have resulted in a reduced chance of sampling rarer genotypes and alleles, and exacerbated the effect of lower quality samples on clustering. Second, in comparison to Dataset 2, samples were up to 27 years older and therefore more likely to have a higher degree of degradation. In summary, our data show that although diploid SNP genotyping is efficient in historical samples, genotyping in duplicated regions is likely to be more sensitive to DNA degradation and should be treated with more caution in quality control and study design.

### DNA pooling and allele frequency estimation (Dataset 2)

The high correlation and repeatability between the estimated and empirical allele frequencies in pooled DNA samples indicate that DNA pooling, even from archived scale material, is a cost-effective solution for estimating sample and population-wide allele frequencies. Accurate estimation of allele frequency may be influenced by the individual DNA quality and concentration within pools, especially in cases where the amount of DNA from particular individuals are under- or over-represented within the sample [22]. Our data indicate that this effect is counteracted by normalisation measures; it is also possible that using a larger number of individuals per pool (>87 individuals in our case) will reduce the influence of a particular individuals on allele frequency estimation [23]. There was more variation between observed and expected DNA frequencies as the minor allele frequency increased in all datasets, showing that allele frequency estimation may be more accurate at loci with lower minor allele frequencies.

As it is unlikely that studies implementing a pooled DNA strategy for allele frequency estimation will genotype all constituent individuals, there are several important considerations when using subsets of samples to determine cluster positions for allele frequency estimation. When sampling smaller subsets of individuals from the full dataset, each increase of 10 individuals significantly improved the accuracy of allele frequency estimation and reducing the amount of stochastic variation affecting the estimates, although the degree of change decreases with each increase in sample size. Sampling genotypes from just 30 individuals meant that more than 95% of pooled samples could be estimated, with a mean difference between the estimated and true empirical allele frequencies of just 0.003 higher than that obtained from the full dataset (Additional file 1: Table S4). Therefore, although we recommend that larger numbers of reference individuals are used, future studies can consider the trade-offs between the number of individuals and the number of pooled samples that can be typed, given particular financial limits for genotyping and the biological question being considered.

### Cluster positioning using subsets of reference individuals (Dataset 2)

When we repeated the analysis of Dataset 2 using smaller subsets of reference individuals for clustering (N = 20 and N = 50), we found that using a lower number of representative samples for the full analysis is likely to introduce some degree of inaccuracy in both cluster positioning and allele frequency estimation. For example, more than 200 loci that failed quality control in the full dataset but were passed in the N = 20 and N = 50 datasets. This may be because problematic loci (such as those which are polymorphic on both paralogues [37]) are more easily detected in the full dataset, but may appear to segregate as normal biallelic loci when clustering with a smaller number of individuals. Furthermore, in the N = 20 dataset, some cluster positions in polyploid regions (MSV-3 and Mono) were placed incorrectly, leading to large discrepancies between the empirical and estimated allele frequencies at a handful of loci (see description of Figure 6). Clustering software, such as Illumina GenomeStudio which is used in the current study, will automatically cluster loci as if they were diploid SNPs, and so 20 individuals may not be enough to visually determine whether or not a locus is an MSV-3, particularly if not all genotypes at a locus are represented. This issue disappeared when the number of individuals used to create the clusters increased to 50, although some allele frequencies inverted between the observed and expected values (i.e. an estimate close to 0 becomes close to 1, and vice versa); this is likely to have arisen in cases where a different allele of a locus is fixed (or almost fixed) in the different paralogues. It should also be noted that a very small proportion of ‘SNP’ loci were misplaced (only 3 out of 3928 in the N = 20 dataset. Therefore, in species with duplicated genomes, a larger representative sample may be required to increase both SNP genotyping rate and the accuracy of allele frequency estimation.

## Conclusions

Although this study has focussed on a single species on a specific genotyping platform, the methods that we have used are applicable to other diploid species, and we have also offered solutions for species with partially duplicated genomes. Overall, we found that SNP genotyping was highly successful in Atlantic salmon scales up to 24 years old, with high genotyping success and low error rates. Furthermore, we demonstrated that allele frequencies could be accurately estimated in pooled DNA obtained from 8–10 year old scales. Our findings open up a new range of opportunities for high throughput SNP analyses using archived material, and further investigations of even older samples may be worthwhile. However, we have also shown that it is imperative to carry out sufficient testing of both DNA condition and SNP genotyping efficiency and to apply strict quality control of samples before embarking on large scale SNP genotyping studies, and that care should be taken to select an appropriate number representative individuals to detect and remove problematic loci and improve the accuracy of allele frequency estimation. Building on previous recommendations for genetic analysis in historical fish scales [12], we make the following additional recommendations for SNP genotyping in historical samples:

### DNA extraction and quality checking

1. Pilot testing should be conducted to identify sample contamination and to determine appropriate thresholds for sample inclusion for SNP genotyping and/or DNA pooling.

2. Effects of sample age on DNA fragmentation and genotyping success should be reliably assessed.

### DNA Pooling

3. Individual samples included in DNA pools should be extracted and normalised using the same methods as all other individuals within the pool.

4. The potential effects of inclusion of DNA samples of varying quality (e.g. of different ages) in pooled DNA samples on accurate allele frequency estimation should be considered.

5. Pools containing larger numbers of individuals are recommended in order to reduce the effects of variation between individuals on allele frequency estimation [23].

### Clustering for SNP genotyping and/or allele frequency estimation

6. Genotyping by clustering should be conducted independently within each genotyping study to account for variation in DNA quantity and quality. Visual inspection is recommended to ensure that clustering is accurate.

7. Reference individuals should come from the same population/samples as the pooled individuals.

8. Individual samples should be genotyped to create informative clusters for accurate allele frequency estimation. In our study, we found that genotyping at least 30 individuals could provide sufficient clustering accuracy for diploid SNP loci.

9. Species with polyploid genomes may require a larger number of reference individuals for allele frequency estimation, to ensure accuracy in cluster positioning.

10. Samples of individual and pooled DNA should be randomised during SNP genotyping to minimise any potential biases from plate position.

11. The estimation of allele frequencies from DNA pools should evaluate the error associated with allelotyping using individually genotyped data.

## Methods

### Sample collection

Atlantic salmon scale samples were collected from wild adult fish captured within the Teno river system in Northern Europe (Norwegian: Tana; 68-70°N, 25-27°E) by local fishermen between 1976 and 2006. Scale samples were air-dried and stored in paper envelopes, which were archived by the Finnish Game and Fisheries Institute at room temperature and humidity in an air-conditioned facility as part of a long-term fisheries monitoring project [39].

### Dataset descriptions, DNA Extraction and DNA quantification

#### Dataset 1: Genotyping in archived scale samples

Archived scales were selected for 16 fish captured in Kevojoki, a tributary within the Teno river system, from the years 1976, 1987, 1996 and 2006 (4 fish per year). For each individual, two independent DNA extractions were carried out on four to five scales using a NucleoSpin® Tissue kit (Macherey-Nagel GmbH, Düren, Germany) in the year 2011. DNA samples were initially normalised to 50 ng/μl using NanoDrop ND-1000 Spectrophotometer (Thermo Scientific). To determine the relative concentrations of DNA of different fragment size ranges for each scale, the normalised samples were prepared using an Agilent DNA 12000 kit and run on an Agilent 2100 Bioanalyzer (Agilent Technologies) at the Finnish Microarray and Sequencing Centre (Turku Centre for Biotechnology, Turku, Finland). DNA quality was categorised as the concentration of DNA (ng/μl) falling within the following five size ranges from low to high molecular weight: 0 - 500 bp, 500 - 1000 bp, 1000 - 5000 bp, 5000 - 17000 bp and > 17000 bp. Samples were checked for contamination by genotyping them on an optimised panel of 15 microsatellites known to be highly polymorphic within Teno Atlantic salmon using the protocol outlined in Vähä et al. (2008); samples were deemed contaminated if more than two alleles were observed at any locus in an individual. SNP genotyping was carried out on each DNA extraction twice, resulting in four replicate genotypes for each individual sample i.e. two for each DNA extraction replicate.

#### Dataset 2: Individual genotyping and allele frequency estimation by DNA pooling

Scales were collected from 530 fish from the Teno river mainstem between 2001 and 2003. DNA was extracted from either one or two scales using a QIAamp DNA mini kit (Qiagen Inc. Valencia, CA, USA) in the year 2011. The DNA concentration of each sample was determined using a NanoDrop ND-1000 Spectrophotometer (Thermo Scientific) and all samples were normalised to 100 ng/μl. Individuals were assigned to one of four DNA pools (N = 87–161 per pool), where 2.5 μl of normalised DNA was transferred from each individual sample into its designated DNA pool. Each DNA pool was independently replicated 3 times (i.e. individual DNA samples were re-pipetted from their source stocks; Figure 1), and replicated a further three times before genotyping, resulting in a total of 9 replicates per DNA pool. Genotyping/allelotyping was carried out on all individual and pooled DNA samples; sample order was randomised to remove possible bias due to variation between genotyping runs.

### Genotyping and quality control

All samples were genotyped at 5568 SNP loci using a modified version of a custom-designed Illumina® iSelect SNP-array previously described in [36,37] (see Additional file 3 for a full list of SNP markers included in the current study). The specific methods and reagents used in this protocol are proprietary, but can be briefly summarised as follows: (i) whole genome amplification; (ii) DNA fragmentation, precipitation and resuspension, (iii) hybridisation to a synthetic DNA probe immobilised on an array and complementary to flanking sequence to one side of the SNP allele, (iv) a single base extension of the probe corresponding to the SNP allele, and finally (v) a technology specific array scanning technology which determines which nucleotide(s) have been incorporated. Individual genotypes were scored using the clustering algorithm implemented in the Illumina® GenomeStudio Genotyping Analysis Module v2011.1, which uses information on the normalised SNP intensity (R) and allelic intensity ratio (Theta) to determine individual sample genotypes. Clustering was carried out excluding individual samples where the raw call rate before quality control was below a certain threshold (0.90 and 0.95 for Datasets 1 and 2, respectively). As a result of historical genome duplication in salmonid genomes [38], some loci show polyploidy and have been classified as multi-site variants [37]. Loci classified as ‘MSV-3′ (where a SNP exists on a single paralogue; N = 873), ‘Mono’ (N = 516, where loci in Lien et al. 2011 were monomorphic) and ‘SNP’ (segregates as a normal diploid SNP, N = 3928) were retained (a combined total of 5317 loci), whereas loci with classifications ‘Unknown’, ‘MSV-5′, ‘PSV’ , ‘Mito’ and ‘Failed’ were discarded (see [40] for further discussion on locus classification). Variation in the quality and quality of DNA samples and hybridisation efficiency between loci can lead to differences in cluster positions between studies [41]. In particular, MSV-3 locus genotypes cluster more tightly and are not always accurately defined by the GenomeStudio software (Additional file 1: Figure S1). Therefore, to ensure genotyping accuracy, clusters for all SNP, Mono and MSV-3 loci were visually inspected in the GenomeStudio software: those clustering incorrectly were either re-clustered by hand or discarded. The software assigns a ‘GenCall’ score to each genotype, which is a measure of the reliability the genotype call based on its position relative to the centre of the genotype cluster (see [42] for more information). Therefore, genotypes with a GenCall score below a certain threshold (0.15 and 0.05 for Datasets 1 and 2, respectively) were discarded; all loci with a call rate of < 0.95 were also discarded. Finally, historic genome duplication could mean that loci which are polymorphic with a low minor frequency on both paralogues may falsely appear to segregate as MSV-3 s or SNPs with a higher frequency of heterozygotes than expected; therefore, loci with a heterozygote excess or deficit of a frequency > 0.1 were discarded.

### Data Analysis

#### Dataset 1: Genotyping in archived scale samples

For each genotyped sample, the proportion of loci genotyped successfully (sample call rate) was calculated. The proportion of loci where two different genotypes were recorded for the same locus in the same individual (genotyping mismatch rate) was also calculated across all four replicates. We then examined the relationships between year, locus call rate, individual mismatch rate, total DNA concentration and percentage of DNA of each size category, fitted as continuous variables within Spearman’s rank correlation tests. All models were implemented in R v2.15.2 [43].

#### Dataset 2: Individual genotyping and allele frequency estimation by DNA pooling

The ‘empirical’ allele frequency for each pooled sample was calculated from individually genotypes of the constituent individuals. The ‘estimated’ *B* allele frequency for each pooled sample at each was calculated within the GenomeStudio software, using information from the normalised theta values relative to the cluster positions for the individual genotypes *AA*, *AB* and *BB*:

(1) $$\mathrm{if}\;\theta_{\mathrm{pool}}\leq {\bar{\theta }}_{{}_{\mathrm{AA}}}\;\mathrm{fre}q_{B}=0\;$$

$$\mathrm{if}\;{\bar{\theta }}_{{}_{\mathrm{AA}}}<\theta_{\mathrm{pool}}<{\bar{\theta }}_{{}_{\mathrm{AB}\;}}\;\mathrm{fre}q_{B}=0.5\left(\frac{\mathrm{\theta pool}-{\bar{\theta }}_{\mathrm{AA}}}{{\bar{\theta }}_{\mathrm{AB}}-{\bar{\theta }}_{\mathrm{AA}}}\right)$$

$$\mathrm{if}\;{\bar{\theta }}_{{}_{\mathrm{AB}}}<\theta_{\mathrm{pool}}<{\bar{\theta }}_{{}_{\mathrm{BB}\;}}\;\mathrm{fre}q_{B}=0.5+0.5\;\left(\frac{\mathrm{\theta pool}-{\bar{\theta }}_{\mathrm{AB}}}{{\bar{\theta }}_{\mathrm{BB}}-{\bar{\theta }}_{\mathrm{AB}}}\right)$$

$$\mathrm{if}\;\theta_{\mathrm{pool}}\geq {\bar{\theta }}_{\mathrm{BB}}\;\mathrm{fre}q_{B}=1$$

Where *freq*_*B*_ is the *B* allele frequency, *θ*_*pool*_ is the Theta value for the pooled sample and ${\bar{\theta }}_{\mathrm{AA}}$, ${\bar{\theta }}_{\mathrm{AB}}$ and ${\bar{\theta }}_{\mathrm{BB}}$ are the mean Theta values for the genotypes *AA*, *AB* and *BB*, respectively. The correlation coefficients (adjusted R^2^) between empirical and estimated allele frequencies were calculated using linear regressions implemented in R v2.15.2. The effect of locus classification (i.e. SNP, Mono or MSV-3), minor allele frequency and cluster position/shape (Illumina’s ’GenTrain Score’ – see Illumina Inc. 2005 for more information) on the mean difference between the empirical and estimated allele frequencies (calculated as the maximum of the two values minus the minimum) was determined by fitting a general linear model with these factors as fixed effects. The repeatability of estimated allele frequency in our dataset was determined using a linear mixed model implemented in the R package MCMCglmm [44] with locus included as a random effect, where repeatability was calculated as the proportion of the total variance explained by all loci. The model was run for 50,000 iterations with a thinning period of 50 and a burn-in of 20,000 iterations, specifying a flat prior and a Gaussian error structure. The model was accepted if the independence of the samples in the posterior distribution (i.e. the autocorrelation) was < 0.1.

#### Dataset 2: Allele frequency estimation using subsets of individuals for reference genotypes

Allele frequencies in DNA pools were re-estimated using mean genotype cluster positions calculated from smaller subsets of constituent individuals, comprising of N = 10, 20, …, 100, 150 and 200 individuals sampled without replacement from the full dataset. Each subset size was sampled 100 times. For every iteration, pooled allele frequencies were estimated relative to the mean theta value of the sampled genotypes using equation 1, and the following values were calculated across all 36 DNA pools and all loci: mean adjusted R^2^; mean difference between all empirical and estimated allele frequencies; and the proportion of pools for which allele frequency could be estimated. Differences between the means values for each size of subset were tested with two-sample t-tests assuming unequal variances.

#### Dataset 2: Re-clustering, genotyping and allele frequency estimation using subsets of representative individuals

The entire analysis was repeated (from GenomeStudio clustering, visual examination and allele frequency estimation) in two single subsets of 20 and 50 individuals randomly selected from the full dataset. The genotypes and estimated allele frequencies determined from these smaller subsets were then compared with the empirical allele frequencies calculated from all 530 individuals. In this analysis, the heterozygote excess/deficit threshold was set at a higher level (to 0.5) to account for inflation of the statistic due to smaller sample sizes.

## Competing interests

The authors declare that they have no competing interests.

## Authors’ contributions

CRP, AV, J-PV, ML and SEJ conceived and designed the study. AV, J-PV, PO, EN, and JE oversaw the collection and provision of scale samples. ML conducted the DNA extraction and quantification in Dataset 1. MPK and SL provided access to the SNP array resources and conducted the SNP genotyping. SEJ analysed the data, and SEJ and CRP wrote the first version of the manuscript. All authors commented on and approved the manuscript.

## Supplementary Material

Additional file 1 The results of all the statistical tests carried out on the temporal study of archived scales, and example plots of Cartesian coordinates used to determine genotypes and the relative position of pooled samples in a ‘normal’ SNP locus and in a multisite variant 3 locus (MSV-3). Click here for file

Additional file 2 DNA fragment sizes and additional information on samples used for the temporal studies of archived scales. Click here for file

Additional file 3 **Information on SNP IDs examined in the current study in relation to previous studies using the custom-designed Atlantic salmon Illumina® iSelect SNP-array **[36,37]**.** Click here for file
