# Supplementary material for: Fish scales and SNP chips: SNP genotyping and allele frequency estimation in individual and pooled DNA from historical samples of Atlantic salmon (Salmo salar)
Source: BMC Genomics. 2013 Jul 3;14:439. doi: 10.1186/1471-2164-14-439 (PMC3716687; doi:10.1186/1471-2164-14-439)
Supplement: Additional file 1 — The results of all the statistical tests carried out on the temporal study of archived scales, and example plots of Cartesian coordinates used to determine genotypes and the relative position of pooled samples in a ‘normal’ SNP locus and in a multisite variant 3 locus (MSV-3). [file 1471-2164-14-439-S1.docx]

**Fish scales and SNP chips: SNP genotyping and allele frequency estimation in individual and pooled DNA from historical samples of Atlantic salmon (*Salmo salar*): Supplementary Document 1**


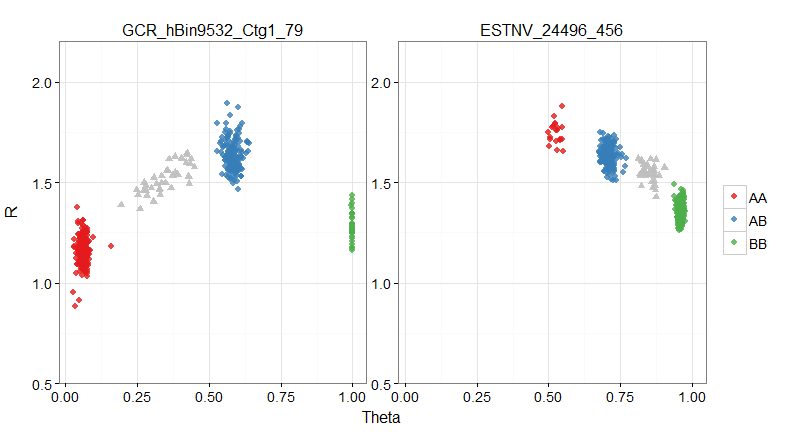


**Figure S1. Example plots of Cartesian coordinates used to determine genotypes (points) and the relative position of pooled samples (triangles) in a normal ‘SNP’ locus (left) and in an ‘MSV-3’ locus (right).** Coloured points represent three genotypes, *AA*, *AB* and *BB*, and grey triangles represent the pooled samples. R and Theta are calculated for each sample from the Illumina GenomeStudio software.

**Table S2. Test statistics for each DNA concentration size classes and Year.**

| **DNA Fragment Size** | **Spearman’s Rho** | **P-value** |
| --- | --- | --- |
| 0 - 500bp | -0.76287 | 3.857 × 10^-07^ |
| 500 - 1000bp | -0.57518 | 5.738 × 10^-04^ |
| 1000 - 5000bp | 0.74773 | 8.729 × 10^-07^ |
| 5000 - 17000bp | 0.84460 | 1.225 × 10^-09^ |
| > 17000bp | 0.08174 | 6.565 × 10^-01^ |
| Combined > 1000bp | 0.77195 | 2.294 × 10^-07^ |

### samples. )allele frequency > 0.05on of matching genotypes and mean GC scoreduals to improve the accuracy of position cluster e


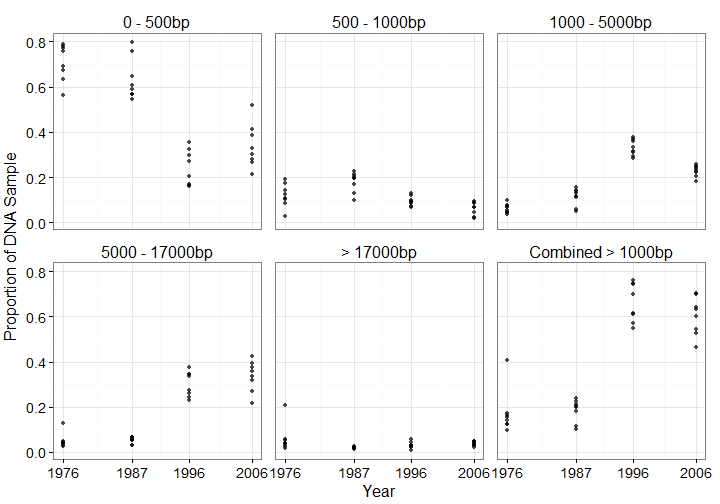


**Figure S2. Correlations between DNA concentration size classes and Year.** Each point indicates an individual DNA extraction. The category “Combined > 1000bp” is the sum of all size classes of 1000bp and greater.

**Table S3. Test statistics for each DNA concentration size class and sample call rate.**

| **DNA Fragment Size** | **Spearman’s Rho** | **P-value** |
| --- | --- | --- |
| 0 - 500bp | -0.7392 | 3.048 × 10^-12^ |
| 500 - 1000bp | -0.2187 | 8.259 × 10^-02^ |
| 1000 - 5000bp | 0.7924 | 6.165 × 10^-15^ |
| 5000 - 17000bp | 0.7290 | 8.511 × 10^-12^ |
| > 17000bp | -0.1313 | 3.011 × 10^-01^ |
| Combined > 1000bp | 0.7386 | 3.26 × 10^-12^ |


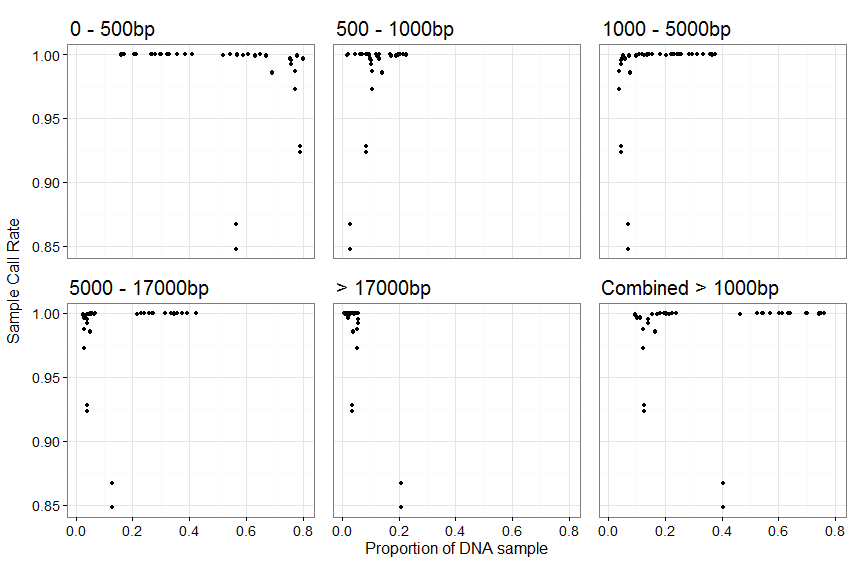


**Figure S3. Correlations between DNA concentration size classes and sample call rate.** Each point indicates an individual genotyping run. The category “Combined > 1000bp” is the sum of all size classes of 1000bp and greater.


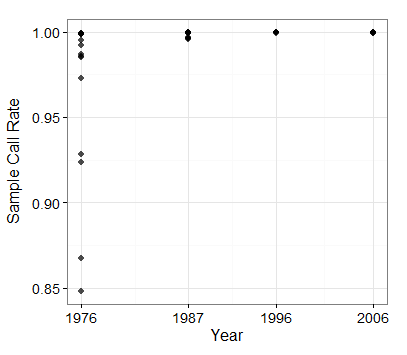


**Figure S4. Correlation between year and sample call rate.** Each point indicates an individual genotyping run. Spearman’s Rho = 0.7387, P = 3.22 x 10^-12^.


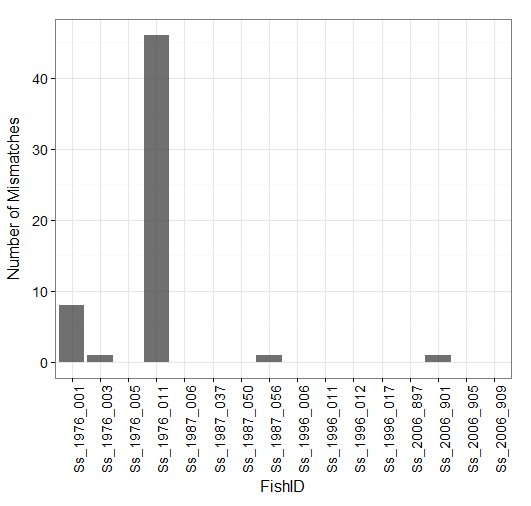


**Figure S5. Number of genotype mismatches per sample over all genotyping runs.** The number after “Ss_” indicates the sampling year.


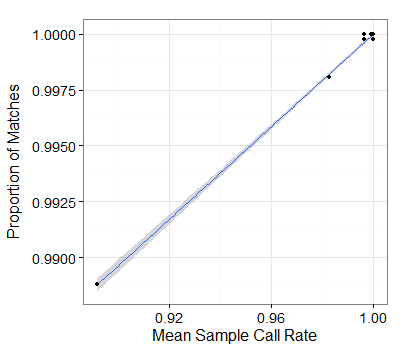


**Figure S6. Correlation between proportion of matching loci and mean sample call rate per individual.** Each point indicates an individual. Spearman’s Rho = 0.7237, P = 0.0015.

**Table S4: Mean estimates of the proportion of pools with estimated allele frequencies, adjusted R^2^ and the mean difference between empirical and estimated allele frequencies using subsets of individuals from the full dataset.** N = 100 samples were taken from each sample size. The values for pool frequencies estimated from the full dataset (514 individuals) are given at the end of the table. Numbers in parentheses are the standard error.

| Number of Individual Genotypes Sampled | Proportion of Pools with Estimated Allele Frequency | | Adjusted R^2^ between Empirical and Estimated Frequencies | | Mean Difference between Empirical and Estimated Allele Frequencies | |
| --- | --- | --- | --- | --- | --- | --- |
| 10 | 0.895 | (5.95E-04) | 0.985 | (3.92E-05) | 0.0267 | (3.54E-05) |
| 20 | 0.948 | (3.79E-04) | 0.986 | (2.50E-05) | 0.0260 | (2.44E-05) |
| 30 | 0.963 | (2.82E-04) | 0.987 | (1.64E-05) | 0.0257 | (1.72E-05) |
| 40 | 0.971 | (2.53E-04) | 0.987 | (1.23E-05) | 0.0256 | (1.44E-05) |
| 50 | 0.975 | (2.38E-04) | 0.987 | (1.36E-05) | 0.0255 | (1.38E-05) |
| 60 | 0.979 | (2.21E-04) | 0.988 | (1.07E-05) | 0.0254 | (1.21E-05) |
| 70 | 0.981 | (2.05E-04) | 0.988 | (9.89E-06) | 0.0254 | (9.70E-06) |
| 80 | 0.982 | (2.09E-04) | 0.988 | (8.95E-06) | 0.0254 | (9.39E-06) |
| 90 | 0.983 | (1.65E-04) | 0.988 | (8.06E-06) | 0.0254 | (9.82E-06) |
| 100 | 0.984 | (1.92E-04) | 0.988 | (8.11E-06) | 0.0254 | (8.43E-06) |
| 150 | 0.987 | (1.46E-04) | 0.988 | (6.35E-06) | 0.0254 | (6.55E-06) |
| 200 | 0.988 | (1.30E-04) | 0.988 | (5.10E-06) | 0.0254 | (5.28E-06) |
| Full (N = 514) | 0.991 | - | 0.988 | - | 0.0253 | - |
